# At What Cost? Trade-Offs and Influences on Energetic Investment in Tail Regeneration in Lizards Following Autotomy

**DOI:** 10.3390/jdb9040053

**Published:** 2021-11-25

**Authors:** James I. Barr, Catherine A. Boisvert, Philip W. Bateman

**Affiliations:** School of Molecular and Life Sciences, Curtin University, Kent Street, Bentley, WA 6102, Australia

**Keywords:** caudal autotomy, fracture plane, growth, lizard, regeneration, reproduction, trade-off

## Abstract

Caudal autotomy, the ability to shed a portion of the tail, is a widespread defence strategy among lizards. Following caudal autotomy, and during regeneration, lizards face both short- and long-term costs associated with the physical loss of the tail and the energy required for regeneration. As such, the speed at which the individual regenerates its tail (regeneration rate) should reflect the fitness priorities of the individual. However, multiple factors influence the regeneration rate in lizards, making inter-specific comparisons difficult and hindering broader scale investigations. We review regeneration rates for lizards and tuatara from the published literature, discuss how species’ fitness priorities and regeneration rates are influenced by specific, life history and environmental factors, and provide recommendations for future research. Regeneration rates varied extensively (0–4.3 mm/day) across the 56 species from 14 family groups. Species-specific factors, influencing regeneration rates, varied based on the type of fracture plane, age, sex, reproductive season, and longevity. Environmental factors including temperature, photoperiod, nutrition, and stress also affected regeneration rates, as did the method of autotomy induction, and the position of the tail also influenced regeneration rates for lizards. Additionally, regeneration could alter an individual’s behaviour, growth, and reproductive output, but this varied depending on the species.

## 1. Introduction

Regeneration, the ‘restoration of a lost body part’, according to Bely and Nyberg [1], is a highly complex physiological process that occurs in many different taxa [2,3,4]. Within the animal kingdom, regenerative abilities vary considerably, having been lost, re-evolved or altered within clades, families, species, and ontogenetic stages [1,3,5,6]. Broadly, regenerative ability and capacity trends decrease evolutionarily with an increase in cellular complexity, transitioning from aquatic to terrestrial lifestyles, causing the development of endothermy and the evolution of an enhanced immune system that favours rapid wound healing through the formation of scar tissue over regeneration [3,5,7,8]. In vertebrates, regenerative ability ranges from the perfect replication of an appendage, as seen in salamanders [9], to the replacement of damaged cells through tissue proliferation, as seen in human livers and intestines [10]. The regrowth of the tail within lizards and tuatara represents a unique case of regeneration since they are the only amniotes capable of regenerating an entire appendage as an adult [4,6,7,9]. All vertebrates possess innate immunity, which is quick and non-specific, as well as an adaptive immunity, which is slower and relies upon antibody production [11]. Lizards lack lymph nodes, a hallmark of mammalian adaptive immunity, and mostly rely on a strong innate immunity to protect themselves from pathogens [9]. It is possible that the compromise between adaptive and innate immunity, with a greater reliance on innate immunity, allows lizards to regenerate, and that a greater reliance on adaptive immunity in mammals reduces their regeneration potential [7,9,12].

### 1.1. Autotomy and Regeneration in Lizards

Caudal autotomy, the ability to drop a portion of the tail, is found in many lizard species and is used as an effective survival tactic when threatened by a predator or conspecific [13,14,15]. Species that rely on caudal autotomy have a series of vertebrae within their tail that have pre-formed planes of weakness, called fracture planes, either within (intra-vertebral) or between (inter-vertebral) the caudal vertebrae [2,13,16]. Intra-vertebral autotomy represents a more ancestral condition compared to inter-vertebral autotomy, with intra-vertebral autotomy seemingly associated with higher neurological control over the process [2,16]. Once a portion of the tail is autotomised, the regenerative process follows three phases: wound healing, blastema formation, and regenerative outgrowth [9]. Immediately following autotomy, sphincters and valves within the caudal arteries of the tail stump constrict to reduce excessive bleeding [2,6,17]. A blood clot forms, and epithelial cells migrate over the wound to form the wound epidermis [9]. After a few days, the wound epidermis thickens and becomes an organised structure called the apical epidermal peg (AEP), with a blastema formation occurring within 10–15 days [9,18]; however, this regeneration latency period can vary dramatically, as shown in Table 1 [5]. The proliferation of cells from the blastema results in the elongation of the regenerating tail [19]. The regenerated tail is not an identical replica of the original, deemed an ‘imperfect regeneration’ [9] and was previously described as a ‘jerry-built’ structure [20]. This is because the regenerated tail is supported by a ridged, semi-ossified continuous cartilage tube rather than the articular vertebrae seen in the original tail [2,20,21]. Once regenerated, the tail can, at least in part, restore biological functionality to individuals [19,22,23,24], although this topic is still under-investigated [25]. For the purpose of this paper, we refer to regeneration as the replacement of a body part with a functional equivalent, with the term ‘lizards’ representing both lizards and tuatara. 

### 1.2. Balancing the Costs of Regeneration Following Autotomy

Lizards’ tails are important for their fitness, playing valuable roles in survival, locomotion, fighting, mate signalling and acquisition, etc., although this is highly variable between species [88,89]. Caudal autotomy is not universal amongst lizards, with the ability lost for certain clades, families, species, and ontogenetic stages [13,14,16,90], partially depending on the importance of the tail or specialisations in question [16,88]. For those species that use caudal autotomy to avoid a predation event, caudal autotomy provides an immediate fitness benefit to the individual through survival but incurs both short- and long-term costs, including physical loss of the tail until regenerated and the energy required for regeneration [2,23]. Goss [4] stated that, for a structure to qualify for replacement, ‘*It must be important enough to be missed when it is gone, but not so vital that an animal cannot survive its loss long enough to grow a replacement*’. Vitt et al. [32] further proposed that, within lizards, the importance of the tail to the individual’s fitness and life history (in the form of future reproductive events) are principal indicators of energy investment in regeneration, and therefore the rate of regeneration. For regeneration to be beneficial to the individual, there should be a net benefit to the individual from costs of energy redirection to regeneration and fitness gain from the regeneration of a ‘functional’ tail, as indicated through its rate of regeneration. Caudal regeneration in lizards has been observed since the 4th century BC by Aristotle; however, understanding the costs and benefits to the individual is highly complex, with a multitude of general, species-specific, and environmental factors influencing regeneration trade-offs. Here, we provide a comprehensive review from the published studies on regeneration rates within lizards and provide a robust discussion on: (1) species-specific, life history and environmental variables that influence regeneration within lizards; (2) the costs and benefits of regeneration in the context of the individual; (3) the difficulty of inter- and intra-species comparisons from previous studies; and (4) provide future recommendations to work toward understanding regeneration within lizards as well as the cost-benefit trade-off in a comparative context.

### 1.3. Literature Search

**Figure 1:**
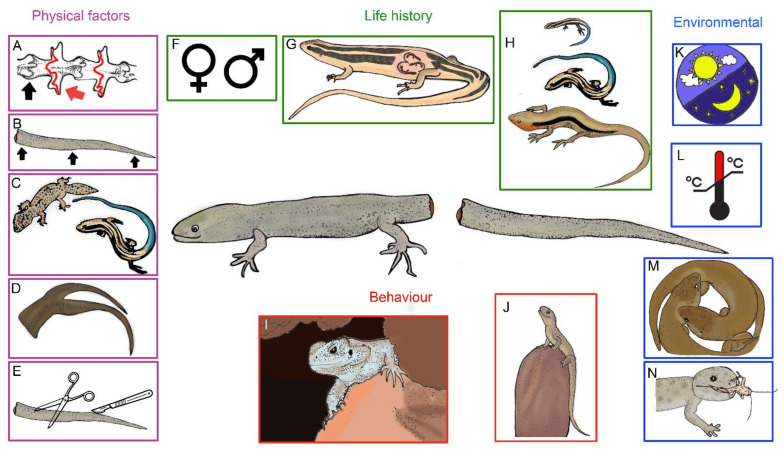
Lizard autotomising tail surrounded by the factors influencing tail regeneration (**A**–**E**) Physical factors: (**A**) Type of autotomy plane—intervertebral (black arrows and lines) and intravertebral (red arrows and lines). (**B**) Position of autotomy. Arrows illustrating proximal, middle, and distal on a tail. (**C**) Importance of tail—examples of tail specialisations for fat storage (gecko: top) and display (skink: bottom). (**D**) Regeneration type—simple or complex, represented by a bifurcated regenerate. (**E**) Type of amputation—autotomy, amputation by scissors or by scalpel. (**F**–**H**) Lifestyle factors: (**F**) Sex. (**G**) Reproduction status represented by a gravid skink with three embryos. (**H**) Developmental stage illustrated by a growth series. (**I**,**J**) Behaviour: (**I**) hiding in a rock crevice or (**J**) basking in the open. (**K**–**N**) Environmental factors: (**K**) Photoperiod. (**L**) Temperature. (**M**) Stressors such as intra- and inter-specific competition represented by intraspecific competition of two skinks fighting. (**N**) Dietary considerations such as the amount of food (ad libitum, limited) and type of food (limited captive diet vs. wild diet) represented by a gecko eating a cricket.

We compiled published records of regeneration rate data within lizards by searching Google Scholar for combinations of ‘caudal’ or ‘tail’ and ‘regeneration rate’ and ‘lizard’, the associated search results that these terms generated, and references gleaned from the citation lists of these papers. Articles were manually searched for regeneration rate information, either directly reported or extracted from figures and tables, or calculated (e.g., amount of tail regenerated in a number of days) from data. As regeneration rates reported or extracted from sources were highly variable, maximum regeneration rates were recorded for the database and included the overall maximum rates for the study period, maximum rate for a specific individual, or, if reported, the maximum regeneration rate for different stages of the regeneration process. Other information extracted from the articles focussed on factors that were known or assumed to influence the regeneration process (Figure 1). These were: family, species, age category (adult or juvenile), point of autotomy (proximal, middle, or distal third), tail status (intact or regenerated), method of autotomy induction (manually induced/pinched or amputated), photoperiod, temperature and latency period until regeneration commenced. Type of fracture plane for species and families were included from additional sources, and the regenerative capability of species in replacing a complete appendage was taken from Appendix 1 of Maginnis [23]. Species names were reported as the most recent classification from The Reptile Database [91]. One exception was *Leiolopisma zealandica*, where a clear reclassification pathway from Barwick [79] could not be established from a complex reclassification history [92].

Photoperiod and temperature were recorded, with photoperiod duration restricted to within 6–18 h of light to be more representative of natural photoperiods, but studies ranging outside these values (e.g., 24L:0D and 0L:24D) were discussed. Studies that were conducted in the field or in outdoor settings were classified as a ‘natural’ photoperiod and temperature if not specified. Regeneration rate information was converted to mm/day for comparison between studies, as was temperature from °F to °C. For studies that investigated the effects of biological compounds on regeneration (i.e., hormones or biological compounds), information for control groups were recorded. Studies that investigated the cost of the regeneration rate, on variables such as reproduction, but did not report extractable information, i.e., those that only reported statistical differences but not regeneration rates, are not included within Table 1 but are discussed in the relevant sections below. 

## 2. Results

Our search results returned 127 records from 65 sources published between 1851 and 2021, from which we extracted data for 56 species across 14 family groups. Regeneration rates varied from 0–4.3 mm/day (Table 1), with most records from captive studies (*n* = 100), sixteen records from field data, and nine that were not specific. Regeneration rate records mostly focussed on adults (*n* = 53), with 21 records from juveniles and 51 not specifying age. The position of autotomy was recorded in sixty-nine records as within the proximal third of the tail, with six records inducing autotomy in either the proximal or middle third, six records in the middle third, five in the distal third and forty-one that were not specific. Autotomy was primarily induced manually by pinching the tail (*n* = 67), through amputation (scissors or scalpel; *n* = 18), and from natural causes (*n* = 18). Regarding tail status, 28 records were derived from observations or studies of individuals with intact tails, 6 had combinations of intact or regenerated tails, two had regenerated tails, and 91 records did not specify tail status. Of the 70 records that reported a photoperiod, they ranged from 6–18 h of light, with 28 studies being classified as ‘natural’ photoperiods. Temperature reports for the records were variable (see Table 1), with records reporting the inclusion of constant temperatures, temperature gradients, as well as studies conducted in ‘room temperature’ or under natural conditions. There were, however, several studies that investigated the effect of different temperatures on regeneration, and these are discussed below (see Section 3.5).

## 3. Discussion

### 3.1. Effects on Regeneration Ability

#### 3.1.1. Type of Autotomy Plane

Most lizards with caudal autotomy have intra-vertebral breakage planes (Figure 1A, red arrow), with the completeness of regeneration seemingly greater in these taxa than in those that have secondarily re-evolved inter-vertebral planes (Figure 1A, black arrow) [16,26]. Despite this, regeneration rates reported for the agamids (the clade within Iguania that has intervertebral autotomy) can be as rapid as for some intravertebral taxa (see Table 1). Although intra-vertebral autotomisers appear to have greater regenerative rates and capacities, it is evident that the type of autotomy does not strictly influence regeneration rates. A recent review of autotomy in agamids by Ananjeva et al. [93] noted that the clade has members that can autotomise and regenerate, autotomise without regeneration (as some snakes do, as well as *Calotes versicolor* [20]), or lack the ability to autotomise at all. Of those that can regenerate, it is not necessarily the speed of regeneration that is reduced but the degree to which the tail can be regenerated to completeness [23,93]. However, even this can be adaptive. Schall et al. [94] noted that *Agama* regenerated lost tails in the form of spiny clubs, particularly in males, which could then be used in intrasexual competitive fights, suggested as potentially conferring a fitness advantage. It should also be noted that the entire loss of autotomy planes, even within the Iguania, does not necessarily mean the loss of regenerative ability—marine iguanas (*Amblyrhynchus cristatus*) with no breakage planes can regenerate tail tissue after damage [95].

#### 3.1.2. Importance of Tail

Tails vary in importance across lizard species and ontogenetic stages [89]. While it seems intuitive that if the tail is important, i.e., has a specialised role such as defence, display or fat storage (Figure 1C), then it will be less likely to be autotomisable, this pattern is not clearly seen across lizards as a whole [88]. Some entire groups, however, such as chameleons, seem to have lost autotomy, as the tail is prehensile [26]. Autotomy is lost ontogenetically in *Corucia zebrata* which also has a prehensile tail [16]. A tail may, therefore, be important both for a particular role, and as an autotomisable part, a good example of this being the coloured tails of young individuals of some skink (*Plestiodon*) species, where the blue tail of juveniles acts to direct predatory attacks away from the body (Figure 1C) [96,97]. However, once these tails are lost, they must be regenerated quickly [32,81,83]. At the other end of the scale is an example such as the slow worm *Anguis fragilis*, a legless lizard living close to the soil–vegetation interface and feeding mainly on slugs: this species shows a very slow and minimal regeneration [30], presumably because the loss of the tail neither impacts foraging nor locomotion enough to necessitate rapid regeneration, nor is autotomy crucial for defence. Lizards face a trade-off between different functions of the tail, and for many species, the tail has vital functions, e.g., locomotion and autotomy. Although the regenerated tail is not an identical copy of the original (see above), it often restores, at least in part, the functionality of the original tail, enough to mitigate long-term fitness effects on the individual [3,23]. Therefore, the speed at which a taxon regenerates its tail is, perhaps, a better indication of the importance of the tail than specialisation, as first suggested by Vitt et al. [32], but this can be influenced by species, life history and environmental factors.

### 3.2. Life History Traits 

#### 3.2.1. Longevity

The priorities of lizards aiming to achieve positive fitness outcomes (e.g., survival, reproductive output) are influenced by their longevity, as well as the importance of the tail and its functionality [23,88,89,98]. A pivotal study by Vitt et al. [32] compared the energetic priorities of short-lived, early-maturing species to long-lived, late-maturing species of lizard in order to evaluate their energetic priorities regarding regeneration and fitness. Vitt et al. [32] concluded that a species’ fitness was increased by favouring rapid regeneration in short-lived, early-maturing species to increase their reproductive output, while long-lived, late-maturing species did not need to prioritise rapid regeneration (see Section 3.4.1). Vitt et al. [32] also hypothesised that the regeneration rate was dependent on the functionality and importance of the tail for each species, where fitness decreases the longer the individual is without a functional tail. In lizards, longevity is correlated to body size, with larger species of lizards having longer lifespans [99]. As larger, more robust lizards tend to rely less on caudal autotomy compared to smaller lizards, the prioritisation of rapid regeneration may not be as important for large lizards as it is for small, short-lived species. Additionally, regeneration rates may follow similar patterns of slower growth rates in long-lived species that live in colder climates (see Section 3.5.1). For example, the regeneration rate of the slow worm, *Anguis fragilis*, a lizard that is known to live for up to 54 years, is exceptionally slow, reaching a maximum of 0.07 mm/day [30]. Bryant and Bellairs [30] further explained that the slow regeneration rate of *A. fragilis* was likely affected by its habits, as it seldom basks and is active over generally lower temperature ranges compared to other species of lizard in the region (see Section 3.5.1). Additionally, the tuatara, *Sphenodon punctatus,* from New Zealand can live for between 60 and 100 years [100,101] and is also active over lower temperatures (between 5 and 13 °C) [102,103]. A very slow regeneration rate of 0.7 cm in 10 months (~0.02 mm/day; Table 1) was recorded for this species [27]. 

#### 3.2.2. Sex

Tail importance, as well as the likelihood of autotomy, can vary between males and females within a species (Figure 1F) [14,88]. The priorities of the sexes and any differential importance of the tail should be reflected in the regeneration rate [2,23,32]. For example, Congdon et al. [46] reported increased regeneration rates for male *Coleonyx variegatus* compared to females, attributing the difference in energy investment to behavioural dimorphism and intra-sexual aggression (Figure 1M). Dial and Fitzpatrick [104] also observed that male *C. brevis* invested more energy into regeneration compared to females, but concluded this was from reproductive priorities (see Section 3.4.1) and not from behavioural dimorphisms. For *Ctenotus taeniolatus*, Taylor [78] found no difference in regeneration rates between males and females, but this may be influenced by the amount of tail autotomised, as lipid distribution varies between sexes in this species. For *Sceloporus olivaceus,* Blair [69] observed that, on average, males had a slightly higher regeneration rate (0.08 mm/day) compared to females (0.05 mm/day), and that this was influenced by age, position of the break in the tail, and season, with similar patterns and influences being observed in the study of *Uta stansburiana* by Tinkle [70] (see Section 3.2.3, Section 3.3.1, and Section 3.5.1).

#### 3.2.3. Age

Regeneration rates vary with the age of the lizard and are likely to reflect their fitness priorities (Figure 1H). Moffat and Bellairs [67] demonstrated that regeneration does not occur until the end stages of the embryonic life of lizards and into post-natal life. As caudal autotomy is primarily an anti-predation strategy, it is unlikely that caudal regeneration would have any benefit to the individual in pre-natal life [105]. When born, however, juvenile lizards generally rely more heavily on caudal autotomy than adults due to their increased risk of predation [13,14]. Although it may be logical that energy prioritisation should focus on regeneration, increasing short-term survival, this can result in larger long-term effects for the individual after they reach maturity (see Section 3.4.2). Vitt et al. [32] hypothesised that growth in juvenile lizards should be favoured to improve their fitness as adults, regarding social rank, territory acquisition, and mating success. Of the four species studied, younger *Coleonyx variegatus*, *Eumeces* (=*Plestiodon*) *skiltonianus*, and *Eumeces* (=*Plestiodon*) *gilberti* invested more energy into body growth, with older individuals prioritising tail regeneration, except in *Gerrhonotus multicarinatus* where energy allocation was similar regardless of age.

Other studies also found regeneration rates to vary between age classes of species, with some studies reporting higher regeneration rates in adults compared to juveniles [74], higher regeneration rates in juveniles compared to adults [69,106], some reporting no difference [70,81], and some reporting high variability within groups [37]. Trade-offs that prioritise growth over tail regeneration can, particularly for juveniles, lead to potentially unmeasured influential effects in some studies. Chapple [74] compared regeneration rates of the metallic skink, *Niveoscincus metallicus*, reporting that juveniles had a regeneration rate of approximately half that of adults (see Table 1) while maintaining comparable growth rates to intact juveniles. However, Chapple [74] also pointed out that, as juveniles are approximately one-third the size of adults, and as regeneration rate is an absolute measure when compared to adults, the proportional rate to body size is relatively rapid. 

### 3.3. Mechanical Effects

#### 3.3.1. Position of Autotomy

Economy of autotomy describes the concept of balancing the short- and long-term costs of caudal autotomy and subsequent regeneration from how much tail is autotomised in a threatening situation (Figure 1B) [13,107]. For example, in an ideal situation, a lizard drops a portion of the tail that is sufficient to distract the threat, allowing it to escape, but minimises the short- and long-term term costs to its fitness from the physical loss of its tail (e.g., lipid reserves, social status, mate acquisition, etc.) and the energy required to regenerate its tail to a point of functionality. The more tail that is lost, the greater the potential fitness effects on the individual; therefore, the quicker the tail is restored to functionality, the lower the fitness hinderance to the lizard [19,23]. The more proximal the autotomy, the quicker the regeneration rate is, in order to minimise the time spent enduring fitness loss. Bryant and Bellairs [30] reported that *Lacerta dugesii* that lost 55–70% of their tails regenerated quicker than those who lost 45–50%. Similarly, in *Uta stansburiana,* Tinkle [70] concluded that breaks in the proximal third of the tail regenerated faster than those in the middle or distal thirds. Tassava and Goss [40] amputated increasing amounts of tail tissue of *Anolis carolinensis* and reported higher regeneration rates when more tail was amputated (Table 1), with maximal regeneration rates peaking at similar time intervals. Abdel Karim [49] observed that *Bunopus tuberculatus* had similar regeneration rates for the first 4–5 weeks regardless of the amount autotomised, after which large divergences were observed, with more proximal amputations having significantly faster regeneration rates.

#### 3.3.2. Mechanism of Inducing Autotomy

In most studies autotomy is induced under experimental conditions by manual pressure, either from the finger and thumb or from callipers stimulating the lizards to contract their tail muscles and shed their tails (Figure 1E). There are, however, studies that amputate the individual’s tail using scissors, a scalpel or a razor blade, as opposed to inducing true autotomy. Some studies have reported that amputation stunted or inhibited regeneration compared to the more natural method of autotomy, although results vary [2]. For example, Jamison [33] investigated this concept by comparing inter-vertebral amputation and naturally induced autotomy for five species of lizard, including two iguanids (*Anolis carolinensis*, *Sceloporus undulatus*), two scincids (*Eumeces* (=*Plestiodon*) fasciatus and *Lygosoma laterale* (=*Scincella lateralis*)) and one anguid (*Gerrhonotus multicarinatus* (=*Elgaria multicarinata*)). Intervertebral amputation severely restricted the caudal regeneration in all species compared to natural autotomy, although it was noted that regeneration was less restricted in the scincids. Jamison [33] concluded that this might result from amputation, reducing the amount of connective tissue exposed, which was linked to the regeneration process. In comparison, other studies described no difference in the regeneration in lizards that had tails amputated compared to autotomised [20,35,59]. Conflicting evidence is presented regarding whether amputation restricts regeneration compared to a more naturally induced method of autotomy (Bellairs and Bryant, 1985). Reported decreases in regenerative capacity from amputation might be the result of the degree of damage to surrounding tissues from the incision and increased inflammation response. A delicate balance between pro- and anti-inflammation is required for regeneration [108,109]. The inhibition of regeneration and potential formation of scar tissue can result from the destruction of nervous tissue, the epithelium, and an increased inflammation response at the wound site [9,18,110]. This may also explain why studies investigating repeated subsequent autotomies observed a reduced regeneration [37,56]. 

#### 3.3.3. Injury and Incomplete Autotomy—Multiple Tails

Previously, the costs and impacts of regeneration to individuals were discussed in the context of normal regeneration ([2,19,23,111], current review). There are, however, instances when the regeneration process goes awry, regenerating additional tails (Figure 1D), usually as a result of a failed or incomplete autotomy event, or if a sufficient wound is inflicted on the tail of the individual [112]. This may have significant implications for life history traits [112]. A study conducted on West Indian Rock Iguana (*Cyclura* spp.) by Hayes et al. [113] across the Bahamian archipelago linked the presence of tail furcations in *Cyclura* with the presence of invasive mammals, mainly the Black Rat (*Rattus rattus*). The review conducted by Barr et al. [112] reported that occurrences of multiple tails, on average, occurred in (±SD) 2.75 ± 3.41% of the natural populations. Although this unusual aspect of regeneration has been recorded for hundreds of years, the costs of this regeneration event, both from an ecological and energy investment context, were not investigated. It is not known how it influences regeneration rates and is mainly limited to anecdotal observations [112]. 

### 3.4. Regeneration Costs on Life History

#### 3.4.1. Reproduction

Following caudal autotomy, reproductive output can be negatively affected for both males and females, both from the physical loss of the tail and energy reallocation into regeneration over reproduction (Figure 1G). The physical loss of a tail can reduce mate pairing and acquisition [61,114,115], and the loss of caudal fat reserves important for vitellogenesis can result in the decreased number and size of young [75,104,116,117], as well as reduced survival from an increased susceptibility to predators [22,118]. As tail functionality can be largely restored once regenerated [23,24], it can be predicted that regeneration should be favoured over reproduction. However, the amount of tail and caudal fat reserves lost, the period when this occurs, i.e., inside or outside of the reproductive season, as well as the longevity of the species (if reproductive output can be balanced over time), and access to food can influence how quickly regeneration will occur, and its potential impacts. Overall, short-lived species have fewer potential reproductive seasons than long-lived species, with lifetime reproductive output likely to be further reduced by costs associated with the tail loss mentioned above. Dial and Fitzpatrick [104] hypothesised that, in short-lived species, if caudal autotomy occurs during the reproductive season, energy investment should favour reproduction over regeneration until the required amount of energy for vitellogenesis has accrued, at which point energy investment should be diverted into regeneration. Long-lived species, however, can focus energy investment into the regeneration and restoration of tail functionality, and immediate costs to reproductive output can be mitigated over future reproductive seasons. This is supported by Bernardo and Agosta [111], where, based on predation risks, regeneration should be prioritised over reproduction to increase the likelihood of survival, more so in long-lived species that have a higher number of potential reproductive seasons compared to short-lived species. Additionally, in their review, Bernardo and Agosta [111] identify that the reproductive costs of caudal autotomy in lizards and salamanders are reduced in those with abdominal fat stores, compared to those that primarily rely on caudal fat storage. In *Uta stansburiana*, a species with a low lipid tail composition [119], tailless females prioritised reproduction and produced heavier young than those with intact tails, at the cost of reduced regeneration [22]. Fox and McCoy [22] concluded that reproductive output was likely maximised through investing in larger, higher-fitness young, by reducing regeneration investment, and by increasing foraging and food intake. In contrast, *Niveoscincus metallicus,* a skink that has fat stores throughout its tail, suffered a reduced litter size during vitellogenesis, regardless of the portion of tail lost [75]. 

#### 3.4.2. Growth

Juvenile lizards invest large amounts of energy into growth during the maturation process (Figure 1H) [2]. Juveniles also rely more on caudal autotomy than do adults, as a result of increased predation risk, and therefore are more likely to drop their tails [14,44,120,121]. As energy reallocation for regeneration is known to compromise other energetically demanding life history traits [23,111], the growth and development of juveniles can, therefore, be compromised. However, studies investigating this produced varying results, with some species showing a decrease in growth post-autotomy [46,122,123], and some species showing no difference in growth [22,44,60,83,119,124]. For example, Webb [44] reported no difference in growth rates of juvenile velvet geckos, *Oedura lesueurii,* with and without tails, with tailless juveniles also investing energy in caudal regeneration; however, a great variation in regeneration rates were reported. Lynn et al. [125] reported no change in body mass for juvenile *Eublepharis macularius*, with and without a tail, but did find that, following autotomy, individuals grew back weightier tails with higher lipid deposits [126]. Ballinger and Tinkle [122] recorded a significant reduction in growth for field populations of tailless *Sceloporus undulatus* and *S. scalaris* hatchlings compared to those with their tails intact, but found no difference for *S. jarrovi* hatchlings maintained in the laboratory and fed ad libitum, concluding that food availability is likely to be a contributing factor (see Section 3.5.3). Althoff and Thompson [119] found no difference in growth between tailed and tailless juvenile *Uta stansburiana* maintained in laboratory settings, and similarly concluded that, in environments free from environmental influences, that a growth trade-off is unlikely to occur (see Section 3.5.4). Fox and McCoy [22] also observed no significant difference in growth for juvenile and adult *U. stansburiana* in field experiments, even with juvenile regeneration occurring more rapidly than in adults. Fox and McCoy [22] concluded that *U. stansburiana* might be more resilient to tail loss, as their tail has minimal lipid stores, and are thus more resistant to the effects of caudal autotomy on life history traits (see Section 3.3.1). 

#### 3.4.3. Altered Behaviour

Following caudal autotomy, and during subsequent regeneration, lizards often exhibit altered behaviour to minimise fitness costs (e.g., risk of predation) until the functional autotomy of the tail is restored (Figure 1I,J) [19,23,24,127]. Many species show a reduction in sprint speed [128], and as such, can compensate by increasing their flight initiation distance (FID), staying close to refuges [129], and reducing activity [115,130,131]. Lizards adjusting their behaviour may simultaneously reduce potential predation risk while promoting caudal regeneration, with behavioural changes likely dependent on the longevity of a species in maximising reproductive fitness. For example, Martín and Salvador [61] showed that following autotomy, male *Lacerta monticola* suffered a reduced social status and access to females. During this time, males increased their body weight, possibly as a result of conserved energy from decreased, intra-sexual, aggressive interactions, with some individuals regaining their status following regeneration. Individuals could also alter their thermoregulatory behaviours, staying closer to refuges and compensating for reduced locomotory capacity, but did not differ in basking frequency or duration, maintaining similar body temperatures [132] and likely suffering few or no negative effects of regeneration associated with thermoregulation (See Section 3.5.1). Additionally, following tail loss in *L. monticola*, males preferred to forage near rocky refuges, adopting a more cryptic strategy, and ingesting a more abundant prey item compared to tailed individuals [133]. Martín and Salvador [61] concluded that, as *L. monticola* has a restricted breeding season, but are relatively long-lived, the prioritisation of regeneration will likely promote long-term reproductive fitness (See Section 3.2.1). However, the extent of behavioural changes and potential compensations can be highly variable, depending on the species [76,134,135,136], degree of tail loss [137,138] and surrounding environment [139].

### 3.5. Environment

#### 3.5.1. Temperature

As ectotherms, lizards’ physiological and metabolic processes are highly dependent on their environmental temperature (Figure 1L) [140,141,142]. As such, lizards will thermoregulate to their preferred body temperatures, balancing the associated costs and benefits [139] to promote regeneration [2]. In captive environments, an increased regeneration rate, as well as a decreased latency of regeneration with higher temperatures, was observed for multiple species (Table 1). For example, Noble and Bradley [71] investigated the effects of temperature on the regeneration of adult *Tarentola mauritanica* in captivity, comparing tail regeneration at a constant temperature of 28 °C or 35 °C, with lizards maintained at the lower temperature regenerating tails at a slower rate. Maderson and Licht [36] investigated the effects of temperature on adult *Anolis carolinensis* at 21 °C and 35 °C, noting a considerable difference in both the time until blastema formation (~36 days vs. ~8 days) and regeneration rate (mean = 0.15 mm/day, max = 0.23 mm/day vs. mean = 0.98 mm/day, max = 1.5 mm/day), respectively. In the scincid *Mabuya* (=*Trachylepis*) *striata*, Magon [86] also observed this pattern during different seasons of the year, with latency to blastema formation being shorter in the warmer seasons and longer in the colder seasons (~6 vs. 14 days) with a maximum regeneration length being achieved more quickly in warmer seasons compared to colder seasons (~6.5 cm in 50 days vs. 90 days). Perhaps one of the most in-depth studies assessing the effect of temperature on regeneration rate was performed by Kurup and Ramachandran [53] on the gekkonid, *Hemidactylus flaviviridis*. Regeneration rates under a 12:12 photoperiod at temperatures of 17 °C, 23 °C, 26 °C, 29 °C, 31 °C, and 33 °C for 30 days were assessed, and there was a longer latency to blastema formation with decreasing temperatures (~6 days at 17 °C–26 days at 33 °C) and an increased regeneration rate with increasing temperatures (mean = 0.60–1.15 mm/day, max = 0.60–2.21 mm/day). This pattern of faster regeneration with increasing temperature is also observed across seasons, with Ramachandran and Ndukuba [55] recording that *H. flaviviridis* regenerated tails faster in the summer season (March-May, mean = 30 °C), followed by the monsoon season (August–October, mean = 26 °C) and slowest in the winter (November–January, mean = 17 °C). This could be linked to the higher metabolic rate associated with higher temperatures; however, some species are metabolically stable over a range of temperatures [143]. Only a few studies included comparisons of ‘natural’ and captive conditions and how they influence regeneration. For example, Maderson and Salthe [37] observed regeneration rates of captive juvenile and adult *Anolis carolinensis* at 32 °C and under ‘natural’ conditions, with both life stages regenerating a larger portion of tail at 32 °C compared to those under natural conditions. This was also observed by Bryant and Bellairs [30], who reported that the regeneration in *Anguis fragilis* was slower for individuals kept outdoors compared to those kept indoors at 27 °C. 

#### 3.5.2. Photoperiod

The photoperiod varies with the time of year and geographic locations, and for reptiles, this can be a significant influencer of both behavioural and physiological processes (Figure 1K) [144,145]. In the context of caudal regeneration, the photoperiod exposure influences the ability of lizards to regenerate their tails. An increased blastema formation and regeneration elongation was observed by Turner and Tipton [38] for *Anolis carolinensis* kept under an 18 h photoperiod compared to those in a 6 h photoperiod, but by week 3–4, regeneration rates were no longer significantly different between treatments. Ndukuba and Ramachandran [146] investigated the effects of eight different light (L):dark (D) photoperiods ranging from 24L:0D to 0L:24D on caudal regeneration in *Hemidactylus flaviviridis* and found that a maximum regenerative capacity was observed in 24 h light treatments, while the lowest growth was observed in 24 h dark treatments., as well as more rapid wound healing and blastema formation. Furthermore, Ramachandran and Ndukuba’s [55] research identified that the regeneration of the tail in *H. flaviviridis* was further reduced in pinealectomized animals but not blinded animals. The pineal gland plays an important role in photosensory reception, circadian activity, and the regulation of melatonin synthesis in vertebrates (Underwood and Goldman, 1987). In lizards, the pineal gland is directly photosensitive through the lizard’s parietal eye [147,148]. Studies have shown that the deprivation of photosensory input, either through pinealectomy [55] or the physical blocking of the third eye photoreceptor [149] inhibits caudal regeneration from the disruption of the natural regulation and release of melatonin [150]. In amniotes, melatonin levels influence the function of the hypothalamic–pituitary–thyroid axis [151,152,153]. The thyroid hormone is essential for the regeneration of muscle and neurons, and the transcriptomic analysis of the regenerating *Anolis* tail shows that a gene for thyroid hormone generation is upregulated in the regenerating tail [154].

#### 3.5.3. Nutrient and Food Availability

Regeneration requires a large amount of energy and can significantly affect both remaining caudal and abdominal fat reserves through an increase in metabolism during regeneration [2,32,104,155]. In the absence of fat stores and/or adequate food, energetic compromise is more pronounced, affecting the life history processes of the individuals [23,111]. For example, food availability, the regularity of feeding, and nutritional value is likely to be different between captive studies and those undertaken in the natural environment (Figure 1N). Ballinger and Tinkle [122] concluded that the difference in the hatchling growth rate observed for *Sceloporus scalaris* hatchlings between field seasons and in the lab is most likely a result of food availability, where growth costs are most likely be minimised in the presence of sufficient food. Similarly, in *Eublepharis macularius*, Lynn et al. [125] found that in restricted food treatments, individuals prioritised energy investment into caudal regeneration over body growth, with individuals maintained on a low-diet regime having a reduced body growth, and those on high-diet regimes having faster regeneration rates for tail mass. Contrary to this, Messner [81] found no difference in the caudal regeneration rates in *Plestiodon obsoletus* maintained in different nutritional food treatments (crickets daily, crickets every other day, and crickets and mealworms daily). 

From the 127 studies compiled in this review, the majority were conducted in captivity and only a small fraction were from natural populations. Of the captive studies, feeding regimes varied from ad libitum to twice weekly, with some studies not reporting their feeding regime. Food items varied, including mealworms, cockroaches, crickets, locusts, weevils, flies, meat, pet food, and bananas. Although it is assumed that the diet provided was adequate and specific to the species requirements, the difference in nutritional intake, for example, mealworms ad libitum vs. meal worms twice weekly, as well as the amount of existing caudal or abdominal fat reserves, may contribute to variation, confounding caudal regeneration rates, as well as inter-study comparisons within and among species [156]. 

#### 3.5.4. Environmental Stressors

Lizards, like most animals, experience biologically induced stress, which is likely to vary depending on their surrounding environment and can have large, adverse effects on their health, compromising their immune systems and decreasing healing ability [157,158]. Stress induces an increase in cortisol, which is linked to reduced wound healing and decreased immunity [159]. Increased predation pressure and lizard densities are known to increase stress from the threat of predation and/or conspecific interactions [160,161,162,163,164]. In the context of caudal regeneration, the effect of stress on regenerative capacity appears to receive little attention, with several studies conducted showing that environmental stress can reduce the regeneration ability of individuals with increased biological stressors. For example, Tsasi et al. [65] investigated the effect of overcrowding stress on *Podarcis erhardii* in captivity, following the induced autotomy of individuals across several populations, varying in predation risk and conspecific densities. Individuals that were housed in ‘crowded’ enclosures (six individuals) had significantly slower regeneration rates compared to individuals housed in a single terrarium. Moreover, individuals from populations with fewer predators and lower lizard densities appeared to be more vulnerable to crowding-induced stress compared to individuals from the population with more predators and higher lizard densities. Exposure to other lizards was demonstrated to induce an increase in blood cortisol levels beyond those induced by autotomy [165]. Cortisol levels are known to play a role in regulating energy allocation [166], which might explain why regeneration and wound healing is reduced, while stress levels are elevated.

In addition to predation and conspecific stress, the parasite load of lizards can dramatically compromise the immune system and health of an individual [167,168,169]. Oppliger and Clobert [68] investigated the effect of parasite load on tail regeneration in *Lacerta* (=*Zootoca*) *vivipara* infected with a protozoan blood parasite (genus *Haemogregarina*) over a 45-day period. Although wound healing duration and specific regeneration rates were not reported, Oppliger and Clobert [68] observed that parasite-free individuals regenerated their tail approximately 1.5 times faster than infected individuals, regenerating 55% of their initial tail length, compared to parasitised individuals who only regrew 35% of their initial tail length. They concluded that the reduction in caudal regeneration was likely a result of an energy trade-off in the maintenance of a strong immune system (as parasite load did not increase during the study) or in other physiological costs, such as reduced haemoglobin and oxygen consumption [170]. 

### 3.6. Future Directions and Recommendations

Regeneration is influenced by the complex interplay of ecological, physiological, environmental, and phylogenetic factors, which are difficult to disentangle [1,2,3,4,5,6,7,8,18,19,23]. Caudal autotomy in lizards is the subject of much research, but rates of regeneration post-autotomy, especially in a comparative sense, receive less attention. In summary, the data on caudal regeneration rates are patchy and limited for lizards and tuatara. Here, we reviewed the available literature that reported on regeneration rates in lizards, highlighting the extensive variability of the regenerative ability between and within species, discussing variables that influence regeneration rates and highlighting the difficulties faced by broader comparative studies. In addition to future studies taking note of the potential importance of the variables discussed to maximise the comparability of studies, we outline some recommendations that we believe would be highly valuable in the future of regeneration.

First, more research focussing on the influential variables from intra- and inter-specific contexts is required. Despite the extensive work conducted on caudal regeneration, data suitable for larger-scale comparisons to uncover phylogenetic trends are still severely lacking. Second, we recommend that, in order to disentangle ecological and evolutionary influencers on regeneration, it would be beneficial to study these trends on a smaller scale. For example, within the family Scincidae in the ‘Egernia’ clade is a grouping of ~50 skinks across seven genera, which include species that have evolutionarily lost fracture planes, species that lose fracture planes ontogenetically, short- and long-lived species, and species that have alternative uses for their tails [171,172]. A clade such as this is small enough for ease of comparison, both between species and on a phylogenetic level, with sufficient variations in morphological and life history traits that allow for a broader understanding on how regeneration rates are influenced. Furthermore, micro-CT is proving to be a valuable, non-destructive, and accurate method for investigating the morphological changes of caudal vertebrae [21,93,173,174] and could be undertaken with live animals, under sedation, to gather more data on regeneration rates. Third, although the effects of stress are known to negatively influence wound healing [159], the type of stressors, as well as the degree of impact are not well represented in lizards in the context of their effect on regeneration. Research into this area would be helpful to better understand the effects of ecological stressors (e.g., conspecifics and parasitism) on regeneration. Finally, the regeneration of abnormal tails is severely underreported and understudied, in the context of the impact on the individual and regeneration rates [112]. In terms of additional tails, furcations of 2–6 furcations were reported as occurring within varying species of lizard [112]. The potential energetic requirements and ecological impacts, both in the short- and long-term, would be of interest in future studies. 

## Figures and Tables

**Table 1:** Regeneration rates reported in reviewed studies for family, species, age, point of autotomy (P = Proximal, M = Middle, D = Distal), tail status (I = Intact/original, R = Regenerated), autotomy induction method (A = Amputated, M = Manual, E = Environmental, NS = Not specific), photoperiod (hours of light, Nat = Natural), temperature range (°C, Nat = Natural), latency period (period until regeneration becomes visible), maximum reported regeneration rate and type of fracture plane (Intra = Intra-vertebral, Inter = Inter-vertebral), regeneration capacity, and sources reviewed. Regeneration capacity from Maginnis [23] (see Appendix 1) describes the ability of the species to regenerate a complete or near complete appendage, with those marked with an asterisk, which represents species in which regeneration occurs, but capacity could not be ascertained. Fracture plane type for families from Arnold [16] and Etheridge [26]. IntraV = Intravertebral, InterV = Inter-vertebral, AbSp = absent in some species, OntLSp = Ontogenetic loss in some species, RstrSp = Positionally restricted to regions of the tail in some species.

Family	Species	Age	Point of Autotomy	Tail Status	Autotomy Induction	Photoperiod Range (h)	Temperature Range (°C)	Latency Period	Max Regeneration Rate Range (mm/day)	Regeneration Capacity	Sources
Agamidae (InterV, AbSp)	*Agama agama*	Adult	-	-	-	-	-	-	0.167	Fair–Good	[27]
	*Calotes versicolor*	-	-	-	A	-	-	-	0	-	[20]
	*Intellagama lesueurii*	-	-	-	-	Nat	Nat	-	0.21–0.77	-	[28]
	*Stellagama stellio*	-	D	-	M	-	-	-	0	Good	[29]
			-	R	E	-	-	-	0		
Anguidae (IntraV, AbSp)	*Anguis fragilis*	Adult	-	I, R	M	-	27, Nat	3–6 weeks	0.05–0.07	-	[30]
	*Anniella pulchara*	-	-	-	-	-	-	~1 week	0.02	Poor	[31]
	*Elgaria multicarinata*	-	P	-	M	-	30	-	0.80–0.84	Fair	[32]
		-	-	-	-	-	22	-	0.5	Fair	[33]
Crotaphytidae (IntraV, AbSp)	*Crotaphytus collaris*	-	-	-	-	-	-	-	0	-	[34]
Dactyloidae (IntraV, AbSp)	*Anolis carolinensis*	Adult	P	I, NS	A	6–18, Nat	15–32	7 days–5 weeks	0.15–1.7	Fair	[35,36,37]
		Adult	-	-	A	6–18	32	6–14 days	0.76–1.34		[38]
		Juvenile	P	I	A	8–16, Nat	15–31.5, Nat	12–14 days	0.24–1.12		[37]
		-	P	-	M	8–18, NS	19–27	~5 weeks	0.05–0.31		[39,40]
			M	-	M	-	19–23	~5 weeks	0.23		[40]
			D	-	M	-	19–30	~5 weeks	0.1–0.40		[40,41]
			-	-	A, M	-	22–32	-	0.12–0.22		[33]
	*Anolis sagrei*	Adult	P	I	M	12	29	-	0.55–0.86	-	[42,43]
Diplodactylidae (IntraV, RstrSp)	*Amalosia lesueurii*	Juvenile	P	-	E	Nat	Nat	-	0.27	-	[44]
	*Dactylocnemis pacificus*	-	-	-	-	-	15–32	-	0.23–0.26	-	[18]
	*Woodworthia maculata*	-	-	-	-	-	20	-	0.14	-	[18]
Eublepharidae (IntraV, RstrSp)	*Coleonyx brevis*	-	-	-	E, NS	-	-	~2 weeks	0.45–1.50	Good	[45]
	*Coleonyx variegatus*		P	-	M	-	30	-	0.60–0.80	Good	[46]
		-	-	-	E	Nat	Nat	-	0.82		[47]
	*Eublepharis macularius*	Adult	P	I	M	-	28–33	-	0.71	-	[48]
Gekkonidae (IntraV, AbSp, RstrSp)	*Banopus tuberculatus*	Adult	P	I, NS	A, M	8–12	27–30	5–7 days	0.14–0.53	Fair	[49,50]
		Adult	M	-	A	8–12	30	5–7 days	0.21–0.40		[49]
		Adult	D	-	A	8–12	30	5–7 days	0.14–0.30		[49]
	*Cyrtodactylus sumonthai*	Adult	-	-	-	Nat	Nat	-	0.88	-	[51]
	*Hemidactylus flaviviridis*	Adult	P	I, NS	M	12, NS	12.4–42.4	~5–26 days	0.28–2.21	Fair	[52,53,54,55]
		-	-	-	-	-	-	-	1.12		[20] as mentioned in [2,56]
	*Hemidactylus garnotii*	-	-	-	E	-	-	-	0.70	Fair	[57]
	*Hemidactylus mabouia*	-	P	I, R	M	12	21–30	~2 weeks	1.1–1.57	-	[58]
	*Hemidactylus turcicus*	Juvenile	D	-	M	-	-	~10 days	0.30	Fair	[59]
	*Paroedura picta*	Juvenile	P	I	M	12	27	1 week	0.75	-	[60]
Lacertidae (IntraV)	*Iberolacerta monticola*	Adult	P	-	-	Nat	-		1.31	Good	[61]
	*Lacertaagilis*	-	-	-	-	-	-	>17 days	0.33–2.0	Good	[62,63]
	*Phoenicolacerta laevis*	-	M	-	A	-	25–27	~8 days	1.0	-	[64]
	*Podarcis erhardii*	Adult	P	I	M	12	25	-	0.70	-	[65]
	*Podarcis muralis*	Juvenile	-	-	-	-	-	-	0.89	Good	[27]
		-	-	-	-	-	27–33	-	0.77		[18]
	*Teira dugesi*	-	P, M	I, R	M	-	27–30	6–10 days	1.3–2.57	-	[30]
	*Timon lepidus*	Adult	P	I	M	12	31	1 week	1.14–4.3	-	[66]
	*Zootoca vivipara*	Adult	-	-	A	-	-	14–21 days	0.67– 0.86	Good	[67]
		Juvenile	P	I	-	-	-	14–21 days	0.46		[68]
Phrynosomatidae (IntraV, AbSp)	*Sceloporus olivaceus*	Adult	P, M	-	E	Nat	Nat	-	0.94–1.05	-	[69]
		Juvenile	P, M	-	E	Nat	Nat	-	1.20–1.57		[69]
	*Sceloporus undulatus*	-	-	-	M	Nat	Nat	-	0.20	Good	[33]
	*Uta stansburiana*	Adult	-	-	E	Nat	Nat	1 week	0.67–0.70	Good	[70]
		Juvenile	-	-	E	Nat	Nat	1 week	0.55–1.0		[70]
Phyllodactylidae (IntraV)	*Tarentola mauritanica*	Adult	P	I	M	-	28–35	-	0.62–0.75	-	[71]
	*Thecadactylus rapicauda*	-	P	-	-	-	-	<1 week	0.47–0.61	-	[72]
Scincidae (IntraV, AbSp, RstrSp)	*Brasiliscincus heathi*	Adult	P	I, R	M	-	25–30	~2 weeks	1.6	Good	[73]
	*Carinascincus metallicus*	Adult	P	I, R, NS	M	14	12–35	<3 weeks	1.18–1.78	-	[74,75]
		Juvenile	P	I	M	14	12–35	<3 weeks	0.61		[74]
	*Carlia jarnoldae*	Adult	P	R	M	Nat	Nat	-	0.60	-	[76]
	*Chalcides ocellatus*	Juvenile	P	I	M	6	18–26.5	-	0.12–0.22	-	[77]
	*Ctenotus taeniolatus*	-	P	-	-	Nat	-	-	0.90–0.98	-	[78]
	*Lampropholis delicata*	-	-	-	-	-	24–28	-	0.60	-	[18]
	*Lampropholis guichenoti*	-	P	-	-	Nat	-	-	0.25–0.33	*	[78]
	*Leiolopisma zealandica*	-	-	-	E	Nat	Nat	~2–3 weeks	0.16–0.37	-	[79]
	*Oligosoma maccanni*	-	-	-	-	-	26–28	-	0.28	-	[18]
	*Ophiomorus streeti*	-	-	-	-	-	-	-	0.09	Poor	[80]
	*Plestidon obsoletus*	Adult	M	-	M	12	-	-	0.49	–	[81]
		Juvenile	M	-	M	12	-	-	0.76		[81]
	*Plestiodon fasciatus*	Juvenile	P	I, NS	M	6–12, Nat	18–33, Nat	8 days	0.39–1.14	Fair–Good	[77,82,83]
		-	-	-	M, E	Nat, NS	22, Nat	-	0.60–0.65		[33,84]
	*Plestiodon gilberti*	-	P	-	M	-	30	-	0.52–0.62	Fair	[32]
	*Plestiodon laticeps*	Juvenile	P	-	M	-	Nat	8 days	0.51	Good	[83]
	*Plestiodon skiltonianus*	-	P	-	M	-	30	-	0.38–0.67	Good	[32]
	*Scincella lateralis*	-	-	-	M, NS	12, NS	21–24	~5–7 days	0.60–2.0	*	[33,85]
	*Trachylepis striata*	Adult	P	I	M	-	10.2–22.8	~6–14 days	0.72–1.69	–	[86]
Sphaerodactylidae (IntraV)	*Sphaerodactylus argus*	-	M	-	M	-	25–30	10–12 days	0.47	Fair–Good	[56]
Sphenodontidae (IntraV)	*Sphenodon punctatus*	Adult	-	-	-	-	-	-	0.02	–	[27]
Xantusiidae (IntraV)	*Xantusia vigilis*	-	P, M	-	-	-	-	-	0.22	Fair–Good	[87]

## Data Availability

The data presented in this study are available on request from the corresponding author.
